# Excitatory Synaptic Transmission Is Differentially Modulated by Opioid Receptors along the Claustrocingulate Pathway

**DOI:** 10.1523/ENEURO.0219-25.2025

**Published:** 2025-08-08

**Authors:** Jacob M. Reeves, Erwin Arias-Hervert, Gracianne E. Kmiec, William T. Birdsong

**Affiliations:** ^1^Neuroscience Graduate Program, University of Michigan, Ann Arbor, Michigan 48109; ^2^Department of Pharmacology, University of Michigan, Ann Arbor, Michigan 48109

**Keywords:** claustrum, cortex, neuromodulators, opioids, recurrent excitation, synaptic transmission

## Abstract

The anterior cingulate cortex (ACC) plays a pivotal role in processing pain and emotion, communicating with both cortical and subcortical regions involved in these functions. The claustrum (CLA), a subcortical region with extensive connectivity to the ACC, also plays a critical role in pain perception and consciousness. Both ACC and CLA express Kappa (KOR), Mu (MOR), and Delta (DOR) opioid receptors, yet whether and how opioid receptors modulate this circuit are poorly understood. This study investigates the effects of opioid receptor activation on glutamatergic signaling in CLA→ACC circuitry using spatial transcriptomics, brain slice electrophysiology, optogenetics, and pharmacological approaches in mice of both sexes. Our results demonstrated that excitatory synaptic transmission generated by the CLA onto Layer 5 pyramidal (L5 PYR) cells in the ACC are reduced by KOR, MOR, and DOR agonists. However, only KOR agonists reduce monosynaptic transmission from the CLA onto L5 ACC PYR cells, highlighting the unique role of KOR in modulating the CLA→ACC pathway. MOR and DOR agonists only reduced slower, longer-latency recurrent excitatory responses. These findings provide new insights into how opioid receptors regulate the claustrocingulate circuit and demonstrate the distinct, receptor-specific modulation of synaptic transmission within this network.

## Significance Statement

This study investigates how opioids regulate brain circuits controlling pain and emotions. The claustrum, a brain region that processes sensory and emotional information, sends signals to the anterior cingulate cortex, a key area for pain perception and emotional regulation. By examining how opioid receptors influence these signals, the study reveals how each receptor type modulates circuit activity in distinct ways. These findings are important because they better our understanding of how different opioid receptor subtypes uniquely control different properties of synaptic transmission and brain circuit function, offering insights for developing targeted treatments for pain, emotional disorders, and opioid use disorder. This work advances our ability to understand and manipulate brain circuits involved in complex behaviors.

## Introduction

The anterior cingulate cortex (ACC) is a critical hub for emotional and pain-related processes (Barthas et al., 2015). As such, the ACC both sends and receives information from cortical and subcortical regions involved in pain perception and emotional regulation (Krieghoff et al., 2011; Rolls, 2019; Shi et al., 2022). Within these pathways, opioid peptides can activate Mu (MOR), Delta (DOR), and Kappa (KOR) opioid receptors to decrease neuronal activity and neurotransmission and ultimately modulate circuit function. Local ACC neurons express MOR and DOR while also receiving MOR-sensitive innervation from thalamic inputs (Mansour et al., 1987; Mansour et al., 1994; Vogt et al., 1995; Birdsong et al., 2019; Zamfir et al., 2023). While it has been shown that the prefrontal cortex receives KOR-sensitive inputs from the basolateral amygdala (Yarur et al., 2023) and the ventral tegmental area (Margolis et al., 2006), no KOR-sensitive inputs into the ACC or KOR-dependent modulation of local excitatory processing have been characterized.

The claustrum (CLA) is a subcortical brain region lying between the insula and the lateral striatum that expresses high levels of KOR, moderate levels of MOR, and low levels of DOR (Tempel and Zukin, 1987; George et al., 1994; Crick and Koch, 2005; Wang et al., 2011; Chen et al., 2020). The CLA exhibits extensive cortical connectivity; the region's activity is associated with processes including pain perception, attention, consciousness, memory, and sleep (Smith et al., 2020; Atlan et al., 2024). The CLA sends dense projections to the ACC and other frontal cortical areas (Jackson et al., 2018; Ährlund-Richter et al., 2019). While the role of the CLA→ACC pathway in pain processing has been highlighted (Xu et al., 2022; Ntamati et al., 2023; Zhang and Zamponi, 2024), whether and how opioids modulate the CLA→ACC pathway is understudied. Several studies have highlighted opioid action in CLA→prefrontal circuits. Chronic social defeat stress attenuated the excitatory output of the CLA→prelimbic cortex circuit through dynorphin/KOR signaling, being critical for depressive-related behaviors in male mice (Wang et al., 2023). Salvinorin A, a KOR agonist, increased functional connectivity between the CLA and ACC in humans suggesting that KOR agonists can modulate CLA→ACC circuitry (Bagdasarian et al., 2024). However, direct physiological studies investigating (1) the effects of KOR activation on CLA→ACC networks, (2) the location of KOR within these networks, and (3) whether MOR and DOR activation also modulates these networks are lacking and are the focus of this study.

Here we employed spatial transcriptomics, brain slice electrophysiology, optogenetics, and pharmacology to characterize opioid receptor modulation along the CLA→ACC pathway in mice. We found that stimulation of CLA inputs in the ACC elicited excitatory transmission with varying latencies and complex waveforms, suggesting CLA inputs elicit both direct and recurrent excitation in the ACC. KOR, MOR, and DOR agonists all reduced excitatory transmission but to varying degrees. When recurrent polysynaptic transmission was blocked, only KOR agonists retained their ability to inhibit CLA→ACC excitatory synaptic transmission. Therefore, monosynaptic inputs from the CLA were regulated by KOR, while MOR and DOR inhibition was restricted to recurrent cortical excitatory circuits. Together, our data show unique roles of KOR, MOR, and DOR in regulating the CLA→ACC pathway. These data illustrate how the claustrocingulate circuit is modulated by opioids and how different opioid receptor subtypes can independently modulate neuronal communication at the circuit level.

## Materials and Methods

### Animals

All procedures were conducted in accordance with the National Institutes of Health guidelines and with approval from the Institutional Animal Care and Use Committee at the University of Michigan. Mice were maintained on a 12 h light/dark cycle and given *ad libitum* access to food and water. C57Bl/6J mice were obtained from Jackson Laboratories. Mice were Postnatal Day (P)40–80 at the time of viral injection P55–110 at the time of brain slice preparation. Mice of both sexes were used.

### Spatial transcriptomics

Publicly available data from the Allen Institute's “Allen Brain Cell Atlas” were used to identify opioid receptor transcripts in the CLA [Allen Brain Cell Atlas (RRID:SCR_024440) https://portal.brain-map.org/atlases-and-data/bkp/abc-atlas; Yao et al., 2023]. The Allen Brain Cell Atlas Use Case Google Colab notebook (https://github.com/rachelhostetler/ABCAtlas_UseCases/blob/main/Plotting_MERFISH_imputed_gene_expression_in_NT_and_region_subset.ipynb) was used to obtain single-cell transcript expression values. The MERSIFH-C57BL6J-638850 with Imputed Genes + Reconstructed Coordinates dataset was used. Brain Slice IDs C57BL6J-638850.52 and C57BL6J-638850.54 were used to obtain expression data results from the CLA bilaterally. The neurotransmitter type, Glut, was selected to isolate glutamate-expressing cells in the CLA, and *oprk1*, *oprm1*, and *oprd1* genes were selected and analyzed.

### Stereotaxic injections

Stereotaxic injections were performed as previously described (Jaeckel et al., 2024) to deliver adeno-associated virus (AAV) to express channelrhodopsin (a gift from Karl Deisseroth produced by UNC Vector Core). Mice were injected bilaterally with an AAV Type 2 encoding channelrhodopsin-2 [ChR2; AAV2-hsyn-ChR2(H134R)-EYFP] targeting the CLA. In total, ∼100 nl of virus was injected into the CLA (A/P, +1.5 mm; M/L, ±2.7 mm; D/V, 3.4–3.6 mm). To target CLA projecting ACC neurons, mice were injected bilaterally with a retro cre-dependent AAV (AAVrg-EF1a-Cre) in the ACC (A/P, +0.75 mm; M/L, ±0.4 mm; D/V, 1.65 mm) and AAV5-EF1a-double floxed-hChR2(H134R)-EYFP-WPRE-HGHpA in the CLA. Approximately 100 nl was injected into both regions.

### Brain slice electrophysiology

Brain slices were prepared 2–3 weeks following injection of ChR2. Mice were deeply anesthetized with isoflurane and decapitated. Brains were removed and mounted for slicing with a vibratome (Model 7000 smz, Campden Instruments). During slicing, brains were maintained at 34°C in carbogenated Krebs' solution containing the following (in mM): 136 NaCl, 2.5 KCl, 1.2 MgCl_2_–6H_2_O, 2.4 CaCl_2_–2 H_2_O, 1.2 NaH_2_PO_4_, 21.4 NaHCO_3_, and 11.1 dextrose supplemented with 5 µM MK-801. The 300 µM coronal sections containing the ACC were made and incubated in carbogenated Krebs' solution supplemented with 10 µM MK-801 at 32°C for 30 min. Slices were then maintained at room temperature in carbogenated Krebs' solution until used for recording. Only one cell was recorded from each slice due to pharmacological drug washes. For ACC recordings, borosilicate glass patch pipettes (Sutter Instrument) were pulled to a resistance of 2.0–3.0 MΩ and filled with a cesium gluconate-low chloride–based internal solution (in mM: 135 cesium gluconate, 1 EGTA, 1.5 MgCl_2_, 10 HEPES (Na), 2 Na ATP, 0.4 Mg GTP, 7.8 Na_2_ phosphocreatine). Slices were placed in the recording chamber and continuously perfused with carbogenated Krebs' solution at 32–33°C. Layer 5 pyramidal (L5 PYR) neurons were identified based on cell morphology. Whole-cell voltage–clamp recordings were made in L5 PYR neurons at −65 mV holding potential to isolate optically evoked excitatory postsynaptic currents (oEPSCs). The liquid junction potential was not corrected for. Cells were dialyzed for 5 min before experimental recordings. A 5 min stable baseline was recorded before drug perfusion. Drugs were perfused for a minimum of 5 min. For TIPPpsi pretreatment experiments (Extended Data Fig. 2-1), either TIPPpsi or aCSF was perfused for 5 min, followed by perfusion of DPDPE with TIPPpsi and DPDPE without TIPPpsi, followed by TIPPpsi for both conditions, respectively. oEPSCs were elicited every 30 s by LED illumination through the microscope objective (Olympus BX51WI) using a transistor–transistor logic-controlled LED driver and a 470 nm LED (Thorlabs). The LED stimulation duration was 1 ms, and the power output was measured using the microscope objective ranging from 0.83 to 5.59 mW. The power output was adjusted to obtain consistent current amplitudes across cells. oEPSCs were recorded before moving to the next drug condition (baseline, agonist, and antagonist). Whole-cell recordings were made with a MultiClamp 700B amplifier (Molecular Devices) digitized at 20 kHz (National Instruments BNC-2090A). Synaptic recordings were acquired using MATLAB WaveSurfer (MathWorks). Series resistance was monitored throughout the recordings, and only recordings in which the series resistance remained <10 MΩ and did not increase >20% were included.

### Analysis of electrophysiology data

Raw data were analyzed using Axograph. For each condition (baseline, agonist, and antagonist), baseline-subtracted sweeps were filtered with a 1 kHz low-pass Gaussian filter and averaged together and plotted. For the agonist and antagonist drug conditions, the ﬁrst four sweeps were omitted from the average to allow for equilibration of drug and washout of drug within the tissue. Peak current amplitude was calculated from the maximum value of the averaged traces and plotted as % oEPSC reduction. Cells that have no connecting line for the antagonist (Figs. 2, 3) either died or had a series resistance too high to include in this study during the antagonist wash. oEPSC onset latency was calculated from the time of 470 nm LED flash to time of 10% rise of oEPSC. For event detection analysis, the first derivative was taken of each sweep and an event amplitude threshold analysis was performed [settings, minimum event separation = 0 (ms); capture baseline = 1 (ms); capture length = 5 (ms); time to peak for typical event = 0.8 (ms); peak measurement interval = 0 (ms)]. The latency was calculated from the time of the light flash, and events were detected from −50–50 ms in relation to the light flash.

### Statistics

Statistical analyses were performed using GraphPad Prism (GraphPadSoftware). Detailed statistical comparisons and descriptions are reported in each respective ﬁgure legend and are presented in detail in Extended Data Figure 1-1. For all comparisons, *n* (number of cells) and *N* (number of animals) are both reported.

## Results

### *Oprk1* exhibits dense expression, *oprm1* exhibits moderate expression, and *oprd1* exhibits low expression in CLA glutamatergic neurons

**Figure 1.**
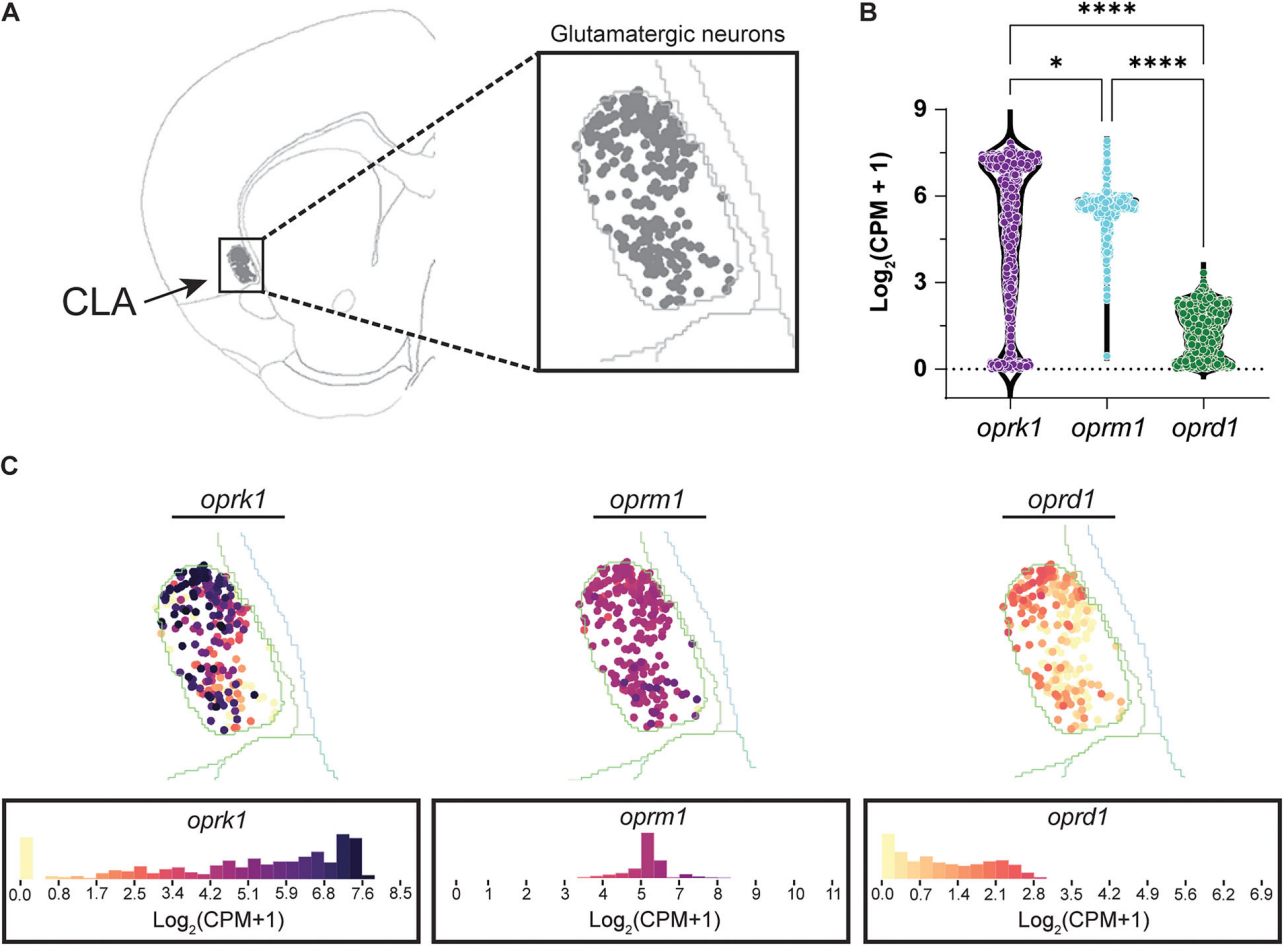
KOR, MOR, and DOR mRNA transcripts are differentially expressed in CLA glutamatergic neurons. ***A***, Example image of a coronal slice of a mouse brain from the Allen Institute's ABC Atlas—whole mouse brain (Brain Slice ID, C57BL6J-638850.52). Gray individual dots are glutamate-expressing neurons in the CLA. ***B***, Summary data of *oprk1*, *oprm1*, and *oprd1* transcripts in CLA glutamate-expressing neurons [*oprk1*, cells, 1,026; *oprm1*, cells, 1,026; *oprd1*, cells, 1,026; *n* (slices) = 2; *oprk1* vs *oprm1*; *p* = 0.0272; *oprk1* vs *oprd1*, *p* = <0.0001; *oprm1* vs *oprd1*, *p* = <0.0001; Kruskal–Wallis test with Dunn's multiple-comparison test]. Each dot represents an individual cell. Additional statistical details are described Extended Data Figure 1-1. ***C***, Example images of *oprk1*, *oprm1*, and *oprd1* expression in glutamate-expressing cells in the CLA. A histogram heatmap of the expression profiles for each transcript is below the example image.

Previous studies have highlighted that the KOR mRNA transcript, *oprk1*, is highly expressed in the rat CLA, while the MOR mRNA transcript, *oprm1*, exhibits moderate expression, and the DOR mRNA transcript, *oprd1*, exhibits low expression (George et al., 1994; Mansour et al., 1994). However, the specific cell types that express these opioid transcripts are not identified in the said studies. In this study, we wanted to observe the expression profiles of *oprk1*, *oprm1*, and *oprd1* in glutamatergic neurons of the CLA. To investigate this, we used publicly available multiplexed error-robust fluorescence in situ hybridization (MERFISH) data from the Allen Institute to identify glutamate-expressing neurons in the CLA and observe the expression values of each opioid receptor transcript. Using the dataset “MERSIFH-C57BL6J-638850 with Imputed Genes + Reconstructed Coordinates” (Allen Brain Cell Atlas; RRID:SCR_024440) from the Allen Brain Cell Atlas, we isolated cells in the CLA that were transcriptionally classified as glutamatergic (Fig. 1*A*). We identified and evaluated slices from this dataset that aligned with our CLA stereotactic injection coordinates (Brain Slice IDs, C57BL6J-638850.52 and C57BL6J-638850.54). Across these glutamatergic CLA neurons, *oprk1* was found to be the most densely expressed opioid receptor transcript, and *oprm1* exhibited consistent moderate expression, while *oprd1* expression was the lowest (in Log_2_(CPM + 1), 25% percentile, *oprk1*, 3.2; *oprm1*, 5.4; and *oprd1*, 0.6; 75% percentile, *oprk1*, 7.1; *oprm1*, 5.8; and *oprd1*, 2.1; Fig. 1*B*,*C*; statistical details for all tests can be found in Extended Data Fig. 1-1). These results suggest that CLA glutamate neurons express high, dense levels of KOR mRNA transcript, moderate levels of MOR mRNA transcript, and low levels of DOR mRNA transcript.

10.1523/ENEURO.0219-25.2025.f1-1 Figure 1-1 Summary table containing all the main effects of the statistical tests that were conducted in this study. Download Figure 1-1, XLS file.

### KOR, MOR, and DOR agonists suppress excitatory synaptic transmission generated by CLA inputs onto L5 ACC PYR cells

**Figure 2.**
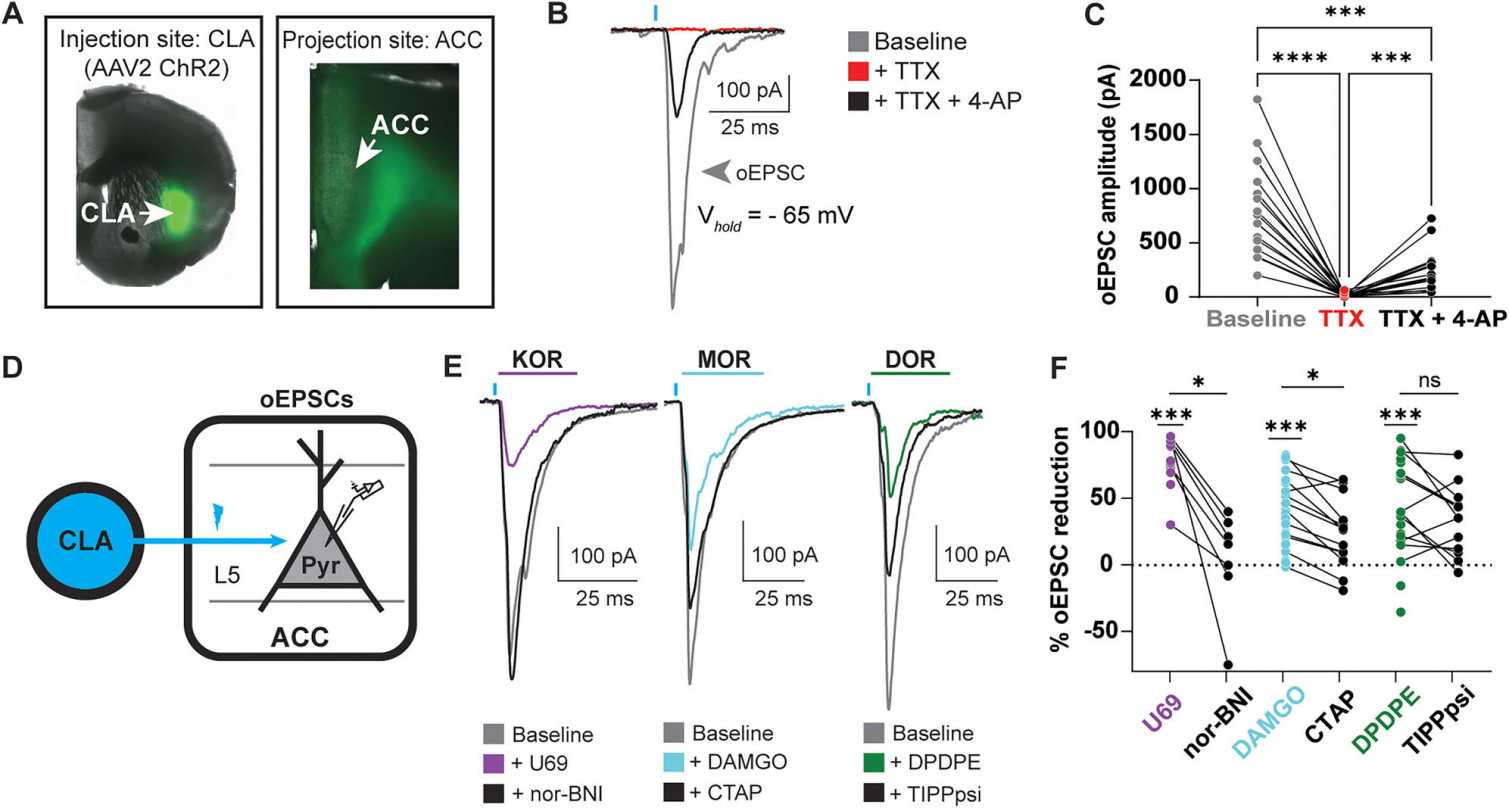
KOR, MOR, and DOR agonists suppress CLA-evoked excitatory synaptic transmission onto L5 ACC PYR cells. ***A***, Channelrhodopsin was virally transduced into the CLA of WT mice; ChR2 expression is seen in CLA terminals innervating the ACC. This was supported by retrograde labeling of ACC-projecting neurons (Extended Data Fig. 2-1). ***B***, Example oEPSC recorded under baseline conditions (gray), TTX (1 µM; red), and TTX (1 µM) + 4-AP (100 µM; black). ***C***, Summary data of oEPSCs of all recordings shown in ***B*** with responses plotted as raw oEPSC amplitude in picoamps (baseline vs TTX; *p* = <0.0001; baseline vs TTX + 4-AP; *p* = 0.0001; TTX vs TTX + 4-AP; *p* = 0.0002; repeated-measure one–way ANOVA with Šídák's multiple-comparison tests; *N* = 9; *n* = 18). ***D***, Schematic of recording oEPSCs from L5 ACC PYR cells evoked by optical stimulation of CLA inputs. ***E***, Example traces of oEPSCs elicited from optical stimulation of the CLA terminals in the ACC during baseline (gray), application of U69 (1 µM, left panel, purple), followed by nor-BNI (1 µM, left panel, black), or application of DAMGO (1 µM, middle panel, cyan) followed by CTAP (1 µM, middle panel, black), or DPDPE (1 µM, right panel, green) followed by TIPPpsi (1 µM, right panel, black). Blue bars, 1 ms of 470 nm light stimulation. While TIPPpsi did not reverse the effect of DPDPE, pretreatment with TIPPpsi (1 µM prevented DPDPE inhibition of the oPISC; Extended Data Fig. 2-2). ***F***, Summary data of oEPSCs from all recordings as shown in ***E*** with responses plotted as a % oEPSC reduction relative to the baseline oEPSC amplitude: baseline versus U69, *p* = 0.0002; *N* = 11; *n* = 16; U69 versus nor-BNI, *p* = 0.0162; *N* = 6; *n* = 7; baseline versus DAMGO, *p* = <0.0001; *N* = 20; *n* = 24; DAMGO versus CTAP, *p* = 0.041; *N* = 10; *n* = 14; baseline vs DPDPE, *p* = 0.0003; *N* = 13; *n* = 23; DPDPE vs TIPPpsi, *p* = 0.2059; *N* = 9; *n* = 13. Mixed-effect analysis with Tukey's multiple-comparison tests conducted with raw amplitudes. *p ≤ 0.05; ***p ≤ 0.001; ****p < 0.0001. Data are described as the mean ± standard error of the mean. Because no differences between sex were observed, data from both sexes are pooled (Extended Data Fig. 2-3).

Previous studies have established that glutamatergic CLA neurons innervate the ACC, with both regions showing dense opioid receptor expression (Tempel and Zukin, 1987; George et al., 1994; Chia et al., 2017, 2020; Ntamati et al., 2023); however, little is known about the opioid sensitivity of the CLA projections to the ACC. Because of the dense KOR expression, moderate MOR expression, and low DOR expression with in the CLA, we hypothesized that excitatory synaptic transmission from CLA to ACC would be reduced by KOR and MOR agonists but not DOR agonists. We tested the opioid sensitivity of ACC-projecting CLA neurons by injecting the blue light-sensitive channelrhodopsin [AAV2-ChR2 (H134R)] into the CLA of wild-type (WT) mice (Fig. 2*A*). Whole-cell patch–clamp recordings were made from L5 ACC PYR cells in acute mouse brain slice preparations. Optical stimulation of CLA terminals in the ACC generated an oEPSC that was blocked by TTX and exhibited variable recovery with TTX + 4-AP (oEPSC amplitude (picoamps, pA); baseline, 832.5 ± 98.8 pA; TTX, 14.02 ± 3.9 pA; TTX + 4-AP, 242.0 ± 43.0 pA; Fig. 2*B*,C). Together these data confirm that the L5 ACC PYR cells receive excitatory synaptic transmission from the CLA.

10.1523/ENEURO.0219-25.2025.f2-1 Figure 2-1 The ACC is innervated by the CLA and not surrounding brain regions. Cre-dependent ChR2 (AAV2-DIO-ChR2 H134R-EYFP was injected in the CLA and retrograde cre-expressing virus (AAVrg-cre) was injected in the ACC. The middle and right images are higher magnification views of the image on the left. Expression is localized to the CLA (middle). Fluorescent CLA terminals are visible in the ACC (right). Download Figure 2-1, TIF file.

10.1523/ENEURO.0219-25.2025.f2-2 Figure 2-2 TIPPpsi pre-treatment blocks DPDPE reduction of CLA evoked oEPSCs onto ACC L5 PYR cells. ***A***, Summary data showing oEPSC amplitude in baseline vs. DPDPE (1  µM) washes in aCSF (black) vs. TIPPpsi (1  µM) (gray) pretreatment conditions (oEPSC amplitude: (aCSF) baseline: 558.1 ± 93.2 pA, DPDPE: 280.3 ± 67.8 pA, N = 3, n = 5; (TIPPpsi) baseline: 667.6 ± 162.0 pA, DPDPE: 607.2 ± 143.3 pA, N = 3, n = 5; (aCSF) baseline vs. DPDPE: *p* = 0.0018; (TIPPpsi) baseline vs. DPDPE: *p* = 0.5045, two-way ANOVA with Šídák's multiple comparisons test). ***B***, Time course of normalized oEPSC amplitude during baseline, DPDPE, and TIPPpsi perfusions in either aCSF or TIPPpsi pretreatment. A schematic is at the top of the graph illustrating the perfusions for each pretreatment condition. Download Figure 2-2, TIF file.

**Figure 3.**
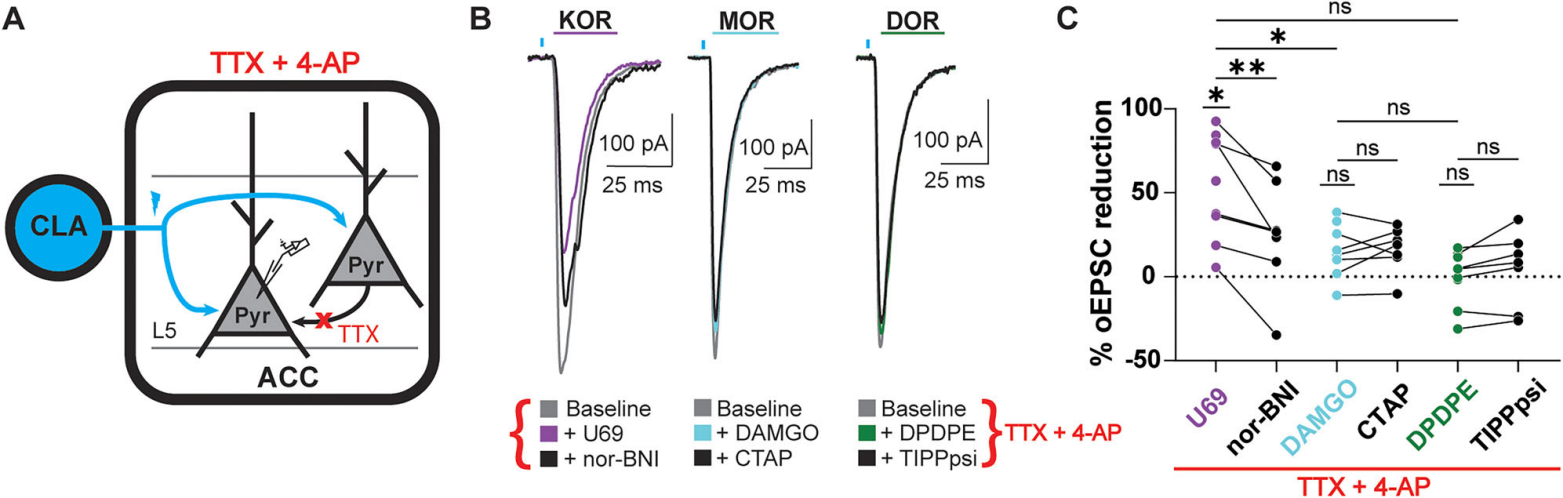
Only KOR activation significantly suppresses monosynaptic oEPSCs from the CLA onto L5 ACC PYR cells. ***A***, Schematic of recording oEPSCs onto L5 ACC PYR cells evoked by optical stimulation of CLA input in the presence of TTX + 4-AP. ***B***, Example traces of oEPSCs elicited by optical stimulation of the CLA terminals in the ACC during baseline (gray), left panel, in U69 (1 µM, purple), and in nor-BNI (1 µM, black), middle panel, in DAMGO (1 µM, cyan) followed by CTAP (1 µM, black), and left panel, DPDPE (1 µM, green) followed by TIPPpsi (1 µM, black) in the presence of TTX + 4-AP. Blue bars, 1 ms of 470 nm light stimulation. ***C***, Summary data of % oEPSC reduction in agonists and antagonists in ***B*** relative to the baseline oEPSC amplitude: baseline versus U69, *p* = 0.022; *N* = 6; *n* = 9; U69 versus nor-BNI, *p* = 0.0041; *N* = 6; *n* = 7; baseline versus DAMGO, *p* = 0.084; *N* = 5; *n* = 8; CTAP, 16.2 ± 5.1%; DAMGO versus CTAP, *p* = 0.353; *N* = 4; *n* = 7; baseline versus DPDPE, *p* = 0.802; *N* = 6; *n* = 9; TIPPpsi, 4.5 ± 8.4%; DPDPE versus TIPPpsi, *p* = 0.264; *N* = 6; *n* = 7; agonist, U69 versus DAMGO, *p* = 0.034; U69 versus DPDPE, *p* = 0.100; DAMGO versus DPDPE, *p* = 0.895. Mixed-effect analysis with Tukey's multiple-comparison tests conducted with raw amplitudes. *p ≤ 0.05; **p ≤ 0.01. Data are described as the mean ± standard error of the mean.

10.1523/ENEURO.0219-25.2025.f2-3 Figure 2-3 There are no sex differences in % oEPSC reduction across opioid receptor subtype. Summary data showing male (closed circles) vs. female (open circles) % oEPSC reduction across U69 (purple), DAMGO (cyan), and DPDPE (green) washes (% oEPSC reduction; U69 males: 77.8 ± 6.2%, U69 females: 89.1 ± 1.7%, U69 males vs. U69 females, *p* = 0.1882. DAMGO males: 42.5 ± 7.4%, DAMGO females: 33.1 ± 7.5%, DAMGO males vs. DAMGO females, *p* = 0.3888. DAMGO males: 54.6 ± 8.7%, DAMGO females: 35.0 ± 10.9%, DAMGO males vs. DAMGO females, *p* = 0.2071; multiple t tests analysis). Download Figure 2-3, TIF file.

Prior studies have shown that the ACC is innervated by the CLA but not by other brain regions surrounding the CLA (Qadir et al., 2018; Atlan et al., 2024). To further verify that our viral injection paradigm results in optical activation of predominantly CLA inputs to the ACC, we injected a retrograde cre-expressing virus (AAVrg-EF1a-Cre) in the ACC and a cre-dependent channelrhodopsin (AAV5-double-floxed-hChR2-EYFP) into the CLA and surrounding areas. Fluorescence expression was only localized to the CLA and not surrounding areas, and CLA axon terminals labeled with YFP were abundant in the ACC (Extended Data Fig. 2-1). These data suggest that oEPSCs recorded in the ACC following injection of AAV2-ChR2 into the CLA are predominantly from excitation of CLA inputs rather than the result of viral leakage into surrounding areas that project to the ACC.

Next, we investigated the opioid sensitivity of excitatory synaptic transmission elicited by optical stimulation of CLA inputs onto L5 ACC PYR cells. The KOR-selective agonist U69,593 (U69, 1 µM) reduced the oEPSC amplitude, and the KOR-selective antagonist nor-BNI (1 µM) largely recovered the oEPSC (% oEPSC reduction; U69, 79.6 ± 4.3%; nor-BNI, 3.5 ± 14.6%; Fig. 2*E*,*F*). The MOR-selective agonist DAMGO (1 µM) reduced the oEPSC amplitude, and the MOR-selective antagonist CTAP (1 µM) recovered the oEPSC amplitude (% oEPSC reduction; DAMGO, 37.4 ± 5.3%; CTAP, 11.4 ± 13.9%; Fig. 2*E*,*F*). The DOR-selective agonist DPDPE (1 µM) reduced the oEPSC amplitude, while the DOR-selective antagonist TIPPpsi (1 µM) did not recover the oEPSC amplitude (% oEPSC reduction; DPDPE, 43.0 ± 7.5%; TIPPpsi, 33.7 ± 7.0%; Fig. 2*E*,*F*). To evaluate that the effect of DPDPE on the % oEPSC reduction was due to DOR inhibition, we pretreated brain slices with TIPPpsi and followed with DPDPE perfusion. Slices that were not pretreated with TIPPpsi exhibited significant reduction of the oEPSC, whereas the oEPSC in slices that were pretreated with TIPPpsi were not significantly reduced (Extended Data Fig. 2-2). There were no sex differences across opioid receptor subtypes, so we combined sexes for this study (Extended Data Fig. 2-3). These data suggest that KOR, MOR, and DOR are functionally present in the CLA→ACC circuit and reduce excitatory synaptic transmission.

### KOR but not MOR and DOR reduce monosynaptic CLA inputs onto L5 ACC PYR cells

Because KOR, MOR, and DOR reduced oEPSC amplitudes, we assessed if all three receptors were functionally localized on monosynaptic CLA inputs onto L5 ACC PYR cells. To assess functional opioid receptor expression on CLA terminals projecting onto L5 ACC PYR cells, we pharmacologically isolated monosynaptic transmission with TTX (1 µM) + 4-AP (100 µM) (Fig. 3*A*). U69 significantly decreased the oEPSC amplitude, and the antagonist nor-BNI recovered the oEPSC amplitude (% oEPSC reduction; U69, 54.6 ± 10.5%; nor-BNI, 24.9 ± 12.5%; Fig. 3*C*). DAMGO and DPDPE did not significantly decrease the oEPSC amplitude (% oEPSC reduction; DAMGO, 15.9 ± 5.8%; DPDPE, −0.2 ± 5.3%; Fig. 3*B*,*C*). These results suggest that KOR but not MOR and DOR are functionally present at significant levels on CLA inputs onto L5 ACC PYR cells.

### oEPSCs onto L5 ACC PYR neurons generated by CLA afferents exhibit both “early” monosynaptic and “late” polysynaptic components

Canonical cortical circuits are modeled to amplify incoming inputs (Douglas and Martin, 2004, 2007; Joglekar et al., 2018; Peron et al., 2020). These inputs can generate an early initial excitatory response followed by a late secondary excitatory response mediated through recurrent excitation generated by local excitatory ACC neurons, driving the amplification process (Harris and Shepherd, 2015; Printz et al., 2023; Vantomme et al., 2025). Opioid agonists could act at multiple sites to reduce excitatory transmission; directly on CLA terminals in the ACC or on local ACC neurons that are involved in recurrent excitation. Because only KOR activation reduced the oEPSC amplitude of monosynaptic CLA inputs in the presence of TTX + 4 -AP, while MOR and DOR activation only inhibited oEPSC amplitude in the absence of TTX + 4-AP, it is possible that MOR and DOR activation selectively inhibits polysynaptic, recurrent excitation driven by the CLA onto L5 ACC PYR cells.

**Figure 4.**
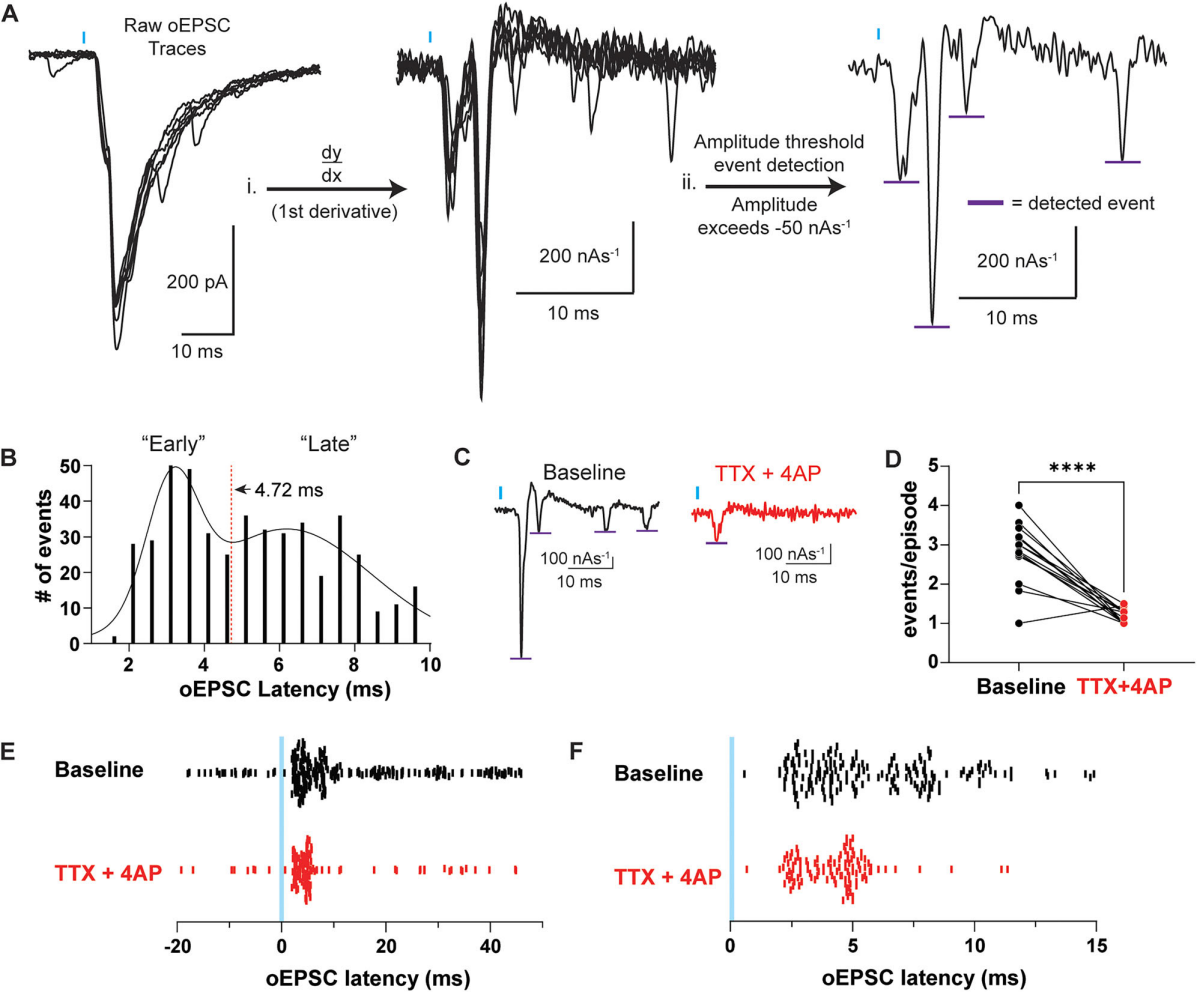
CLA-evoked oEPSCs onto L5 ACC PYR neurons exhibit an early monosynaptic and late polysynaptic component. ***A***, Workflow, ***i***, differentiating the raw voltage-clamp waveform to obtain instantaneous oEPSC slope measurements, ***ii***, event detection analysis with a −50 nA*s^−1^ event threshold. Purple bars are the maximum slope of the detected events. ***B***, Histogram of the maximum slope of oEPSC detected events relative to optical stimulation (oEPSC latency) (*N* = 25; *n* = 52; events detected, 474). ***C***, Example traces of the first temporal derivative voltage-clamp oEPSC waveforms during the baseline (black) and TTX (1 µM) + 4-AP (100 µM; red). Purple lines indicate detected events. ***D***, Comparison of the events/episode in the baseline and TTX + 4-AP conditions (baseline vs TTX + 4-AP; *p* = <0.0001; paired *t* test). ***E***, A plot of events detected in the baseline and TTX + 4-AP conditions from −20 to 50 ms relative to optical stimulation. Each line represents a single event. ***E***, A plot of detected events, as seen in ***F***, from 0 to 10 ms. Blue bars, 1 ms of 470 nm light stimulation. ****p < 0.0001. Data are represented as the mean ± standard error of the mean.

To investigate this, we deconvolved the oEPSC by separating each oEPSC into discrete synaptic events using the first temporal derivative of the voltage-clamp waveform. The derivative of each oEPSC waveform was calculated, and an event detection analysis was carried out (Fig. 4*A*). Baseline events detected from 0 to 10 ms from the time of optical stimulation were binned and plotted (Fig. 4*B*). The data were best fit with a double Gaussian function, indicative of at least two oEPSC populations. The trough of the double Gaussian curve (4.72 ms) was used to divide the detected oEPSC events into “early” and “late” onset oEPSC event populations. To determine whether the late component was likely due to polysynaptic recurrent excitation, we isolated the monosynaptic oEPSC by again recording in TTX and 4-AP (Fig. 4*C*). oEPSC events derived from baseline oEPSCs averaged 2.8 ± 0.2 events/episode which significantly decreased to 1.2 ± 0.1 events/episode in TTX + 4-AP (Fig. 4*D*). Most of the remaining events in TTX + 4-AP fell in the early time window (Fig. 4*E*,*F*). As hypothesized, these results suggest that CLA inputs onto L5 ACC PYR cells generate both an early component (<4.72 ms) in which most monosynaptic oEPSC events fell and a late component (>4.72 ms) in which most polysynaptic oEPSC events fell.

### KOR alters release frequency and synaptic conductance regardless of event latency; however, MOR and DOR do so in a latency and receptor-dependent manner

**Figure 5.**
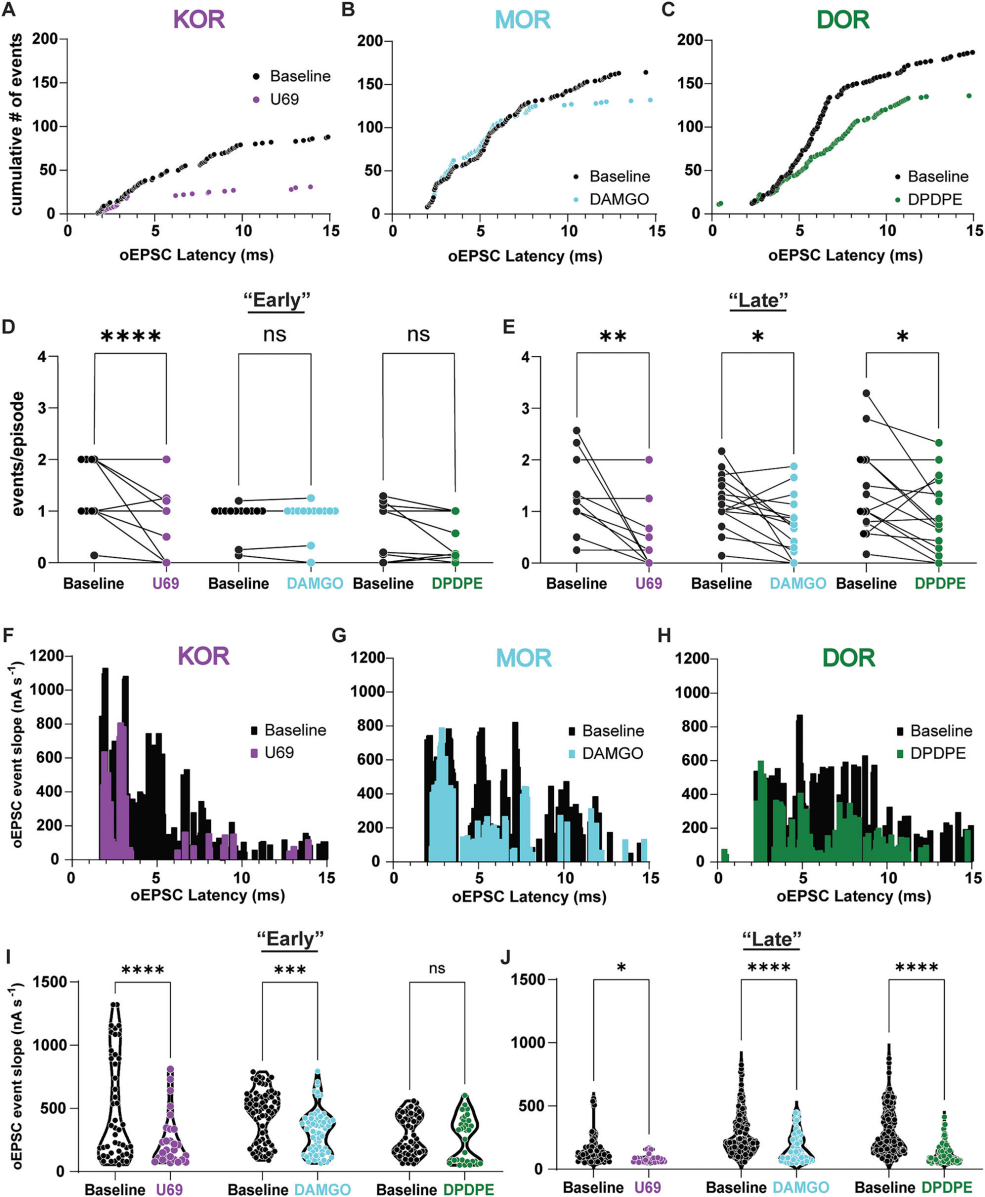
KOR modulates release frequency and synaptic conductance independently of event latency, whereas MOR and DOR exert their effects in a latency- and receptor-dependent manner. ***A–C***, Cumulative distribution of oEPSC events for the baseline (black) and U69 (purple), baseline (black) and DAMGO (cyan), and the baseline (black) and DPDPE (green), respectively. ***D***, Summary data of early oEPSC events/episode for U69, DAMGO, and DPDPE washes compared with baseline conditions (early oEPSC events/episode; baseline vs U69, *p* = <0.0001; *n* = 11; baseline vs DAMGO, *p* = 0.9946; *n* = 12; baseline vs DPDPE, *p* = 0.1893; *n* = 11; two-way ANOVA with Tukey's multiple-comparison test). ***E***, Summary data of late oEPSC events/episode for U69, DAMGO, and DPDPE washes compared with baseline conditions (late oEPSC events/episode; baseline vs U69, *p* = 0.0015; *n* = 10; baseline vs DAMGO, *p* = 0.0183; *n* = 16; baseline vs DPDPE, *p* = 0.0300; *n* = 15; two-way ANOVA with Tukey's multiple-comparison test. ***F–H***, Histogram of the oEPSC event slope vs oEPSC latency for events detected in the baseline (black) and U69 (purple), DAMGO (cyan), or DPDPE (green) washes, respectively. ***I***, Summary data of early oEPSC event slope U69, DAMGO, and DPDPE washes compared with baseline conditions [early oEPSC event slope; baseline (47 events) vs U69 (27 events), *p* = <0.0001; baseline (72 events) vs DAMGO (65 events), *p* = 0.0002; baseline (53 events) vs DPDPE (33 events), *p* = 0.1893; two-way ANOVA with Tukey's multiple-comparison test]. ***J***, Summary data of late oEPSC event slope for U69, DAMGO, and DPDPE washes compared with baseline conditions [late oEPSC event slope; baseline (63 events) vs U69 (29 events), *p* = 0.0216; baseline (117 events) vs DAMGO (68 events), *p* = <0.0001; baseline (149 events) vs DPDPE (94 events), *p* = <0.0001; two-way ANOVA with Tukey's multiple-comparison test]. *p ≤ 0.05; ****p < 0.0001. Data are represented as the mean ± standard error of the mean.

To address the hypothesis that MOR and DOR might selectively reduce local ACC recurrent excitation, we examined the effects of opioid agonists on the deconvolved early and late oEPSC event populations. The cumulative distribution of optically evoked events before and after U69 application revealed a significant decrease in the number of optically evoked events at both early and late timepoints (Fig. 5*A*). However, both DAMGO (Fig. 5*B*) and DPDPE (Fig. 5*C*) selectively decreased the number of late but not early events. To quantify this observation, events were grouped into “early” (<4.72 ms) and “late” (>4.72 ms; as in Fig. 4*B*) oEPSC events, and the effects of U69, DAMGO, and DPDPE were compared. For the early oEPSC events, only U69 significantly decreased the number of events/episode compared with the baseline (early oEPSC events/episode, baseline, 1.3 ± 0.2; U69, 0.6 ± 0.2; baseline, 0.9 ± 0.1; DAMGO, 0.9 ± 0.1; baseline, 0.7 ± 0.2; DPDPE, 0.5 ± 0.1; Fig. 5*D*). For the late oEPSC events, U69, DAMGO, and DPDPE significantly decreased the events/episode compared with the baseline (late oEPSC events/episode, baseline, 1.3 ± 0.2; U69, 0.5 ± 0.2; baseline, 1.2 ± 0.1; DAMGO, 0.7 ± 0.2; baseline, 1.4 ± 0.2; DPDPE, 0.9 ± 0.2; Fig. 5*E*).

Next, we compared the maximum slope of each oEPSC event across conditions, which are related to synaptic conductance (Johnston and Wu, 1994). In this instance, U69 and DAMGO, but not DPDPE, significantly decreased the average oEPSC event slope in early oEPSCs compared with that in the baseline (early oEPSC event slope; baseline, 474.2 ± 59.7 nA*s^−1^; U69, 253.4 ± 40.1 nA*s^−1^; baseline, 451.3 ± 23.7 nA*s^−1^; DAMGO, 295.4 ± 25.5 nA*s^−1^; baseline, 286.8 ± 21.2 nA*s^−1^; DPDPE, 268.3 ± 31.0 nA*s^−1^; Fig. 5*I*), suggesting MOR may have a role in attenuating early oEPSC events. For the late oEPSCs, the average oEPSC event slope was significantly decreased by U69, DAMGO, and DPDPE compared with the baseline (late oEPSC event slope; baseline, 148.0 ± 13.4 nA*s^−1^; U69, 84.2 ± 6.3 nA*s^−1^; baseline, 273.0 ± 15.5 nA*s^−1^; DAMGO, 175.8 ± 13.7 nA*s^−1^; baseline, 287.6 ± 14.2 nA*s^−1^; DPDPE, 124.0 ± 7.9 nA*s^−1^; Fig. 5*J*). These data suggest that KOR inhibits CLA inputs to the ACC which results in little to no recurrent excitation, while MOR and DOR both preferentially reduce the late polysynaptic component of the oEPSC. However, MOR had an effect on the early oEPSC event slope but not the early events/episode. This effect could suggest either presynaptic (less glutamate release) or a postsynaptic (less AMPA activation or a shunting GIRK conductance) effect of MOR activation; however, no changes in holding current or input resistance were observed. Altogether, these results support the idea that KOR predominantly inhibits CLA inputs while MOR and DOR predominantly suppress polysynaptic recurrent excitation in the CLA→ACC pathway.

### MOR and DOR reduce excitation in a latency-dependent manner, whereas KOR reduces all excitation regardless of latency

**Figure 6.**
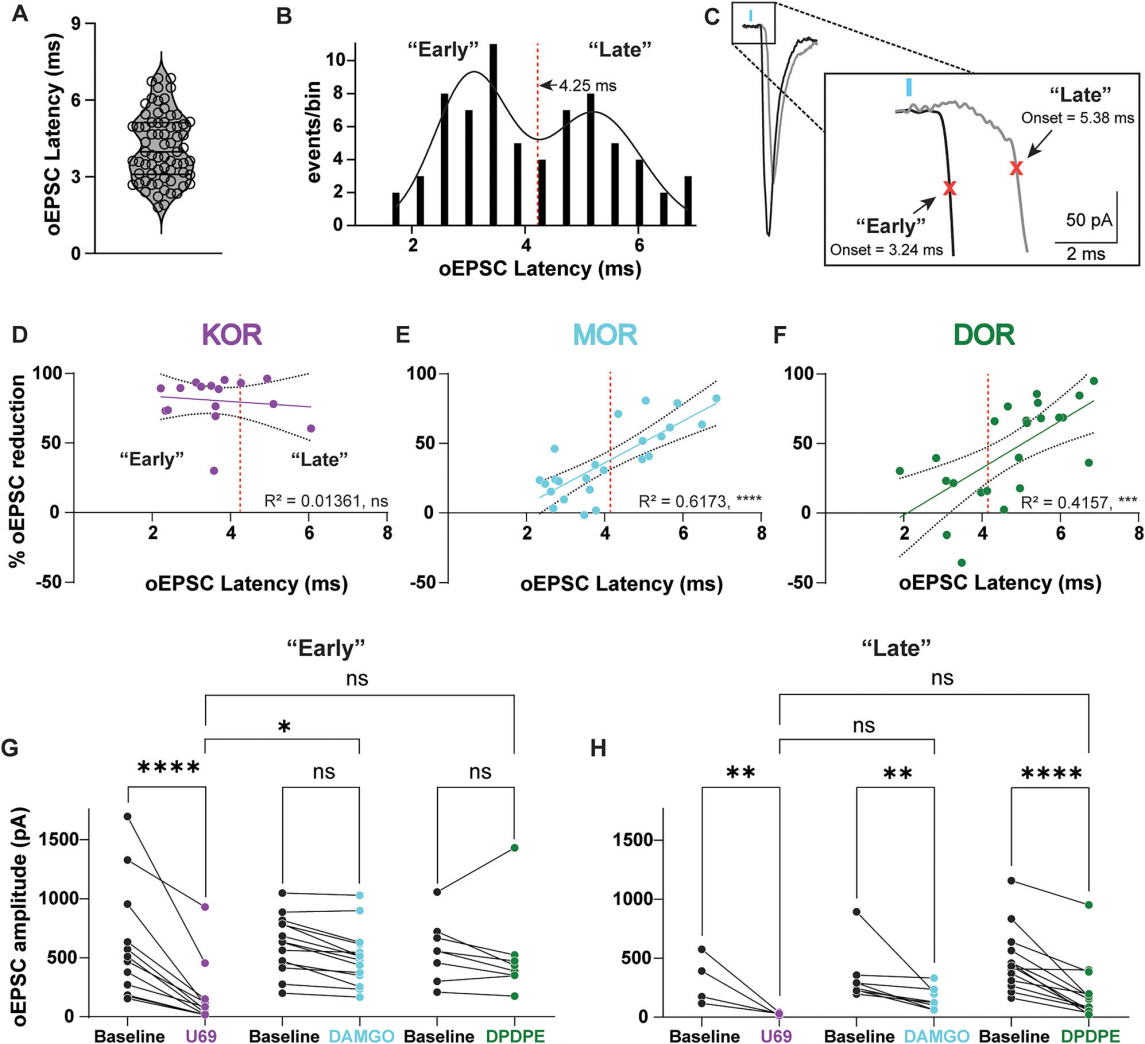
oEPSC onset latency correlates with oEPSC reduction by MOR and DOR but not KOR agonists. ***A***, A violin plot of individual baseline oEPSC latencies (*n* = 69). ***B***, Histogram of oEPSC onset latency values as shown in ***A***. The red line divides “early” and “late” events. ***C***, Example traces of a “early” (black) and “late” oEPSC (gray). Traces are from two different cells. ***D***, A plot of the % oEPSC reduction by U69 relative to oEPSC latency. There was no correlation between the % oEPSC reduction by U69 and oEPSC onset latency (simple linear regression, *R*^2^ = 0.01361; *p* = 0.6698). ***E***, A plot of the % oEPSC reduction by DAMGO relative to oEPSC latency. There was a significant correlation between the % oEPSC reduction by DAMGO and oEPSC onset latency (simple linear regression, *R*^2^ = 0.6173; *p* = <0.0001). ***F***, A plot of the % oEPSC reduction by DPDPE relative to oEPSC latency. There was a significant correlation between the % oEPSC reduction by DPDPE and oEPSC onset latency (simple linear regression, *R*^2^ = 0.4157; *p* = 0.0009). ***G***, Summary data of early oEPSC amplitudes across baseline and agonist washes (U69, DAMGO, and DPDPE; baseline vs U69, *p* = <0.0001; *n* = 12; baseline vs DAMGO, *p* = 0.0630; *n* = 14; baseline vs DPDPE, *p* = 0.5324; *n* = 8. ***H***, Summary data of late oEPSC amplitudes across the baseline and agonist washes (U69, DAMGO, and DPDPE). Baseline vs U69, *p* = 0.0044; *n* = 4; baseline vs DAMGO, *p* = 0.0098; *n* = 8; baseline vs DPDPE, *p* = <0.0001; *n* = 14. *p ≤ 0.05; **p ≤ 0.01; ***p ≤ 0.001; ****p < 0.0001. Dashed red line, 4.25 ms. Data are represented as the mean ± standard error of the mean.

Our data suggest that KOR reduces early monosynaptic CLA inputs onto L5 ACC PYR cells, whereas both MOR and DOR reduce late recurrent excitation generated by CLA inputs. To further support this, we next determined whether the timing-dependent opioid receptor inhibition could be resolved without deconvolution of oEPSCs into individual events. To investigate this, we used the raw oEPSC data for analysis seen in Figure 2*F*. First, we measured the oEPSC onset latency from raw traces under baseline conditions for all recordings to determine how variable oEPSC onset latency was. Similar to the findings from the derivative analysis in Figure 4*B*, the oEPSC latency histogram revealed a bimodal distribution of oEPSC onset latency that was best fit with a double Gaussian function, suggesting two different oEPSC populations identified based on onset latency (Fig. 6*A*,*B*). The trough of the double Gaussian best fit occurred at 4.25 ms. Therefore, oEPSCs were grouped into “early” oEPSC's (onset values <4.25 ms) and “late” oEPSC's (onset values >4.25 ms; Fig. 6*B*,*C*). If KOR acts on monosynaptic CLA inputs while MOR and DOR act on polysynaptic inputs, we hypothesized that there would be a positive correlation between oEPSC latency and MOR- and DOR-mediated inhibition of the oEPSC. However, because KOR acts on monosynaptic CLA inputs, both early and late oEPSCs should be suppressed due to inhibiting CLA terminals which are then unable to activate recurrent excitation, leading to no correlation between onset latency and oEPSC inhibition.

As hypothesized, there was no significant correlation between oEPSC onset latency and % oEPSC reduction by U69 (Fig. 6*D*). However, both DAMGO (Fig. 6*E*) and DPDPE (Fig. 6*F*) exhibited a significant correlation between oEPSC onset latency and % oEPSC reduction. The oEPSC data were then grouped and analyzed by “early” and “late” oEPSCs split by 4.25 ms (based on Fig. 6*B*), and the effects of drugs were compared across onset latency. U69, but not DAMGO nor DPDPE, significantly reduced the peak amplitude of early-onset oEPSCs [early-onset % oEPSC reduction (oEPSC amplitude); baseline, 610.0 ± 140.2 pA; U69, 166.7 ± 77.7 pA; baseline, 617.3 ± 8.8 pA; DAMGO, 502.9 ± 63.8 pA; baseline, 564.6 ± 93.2 pA; DPDPE, 515.0 ± 136.0 pA; Fig. 6*G*]. For the late-onset oEPSCs, U69, DAMGO, and DPDPE, all significantly reduced the oEPSC peak amplitude compared with the baseline [late-onset % oEPSC reduction (oEPSC amplitude); baseline, 174.5 ± 51.2 pA; U69, 28.5 ± 5.2 pA; baseline, 340.8 ± 80.9 pA; DAMGO, 157.0 ± 33.6 pA; baseline, 480.8 ± 69.7 pA; DPDPE, 209.3 ± 65.0 pA; Fig. 6*H*].

Together, these results suggest that opioid receptor modulation affects multiple pathways along the CLA→ACC circuit, while MOR and DOR activation preferentially suppresses late oEPSCs and KOR activation drastically suppresses all oEPSCs. These results are consistent with KOR being expressed on CLA terminals in the ACC, while MOR and DOR are expressed on local ACC neurons that modulate recurrent excitation.

## Discussion

**Figure 7.**
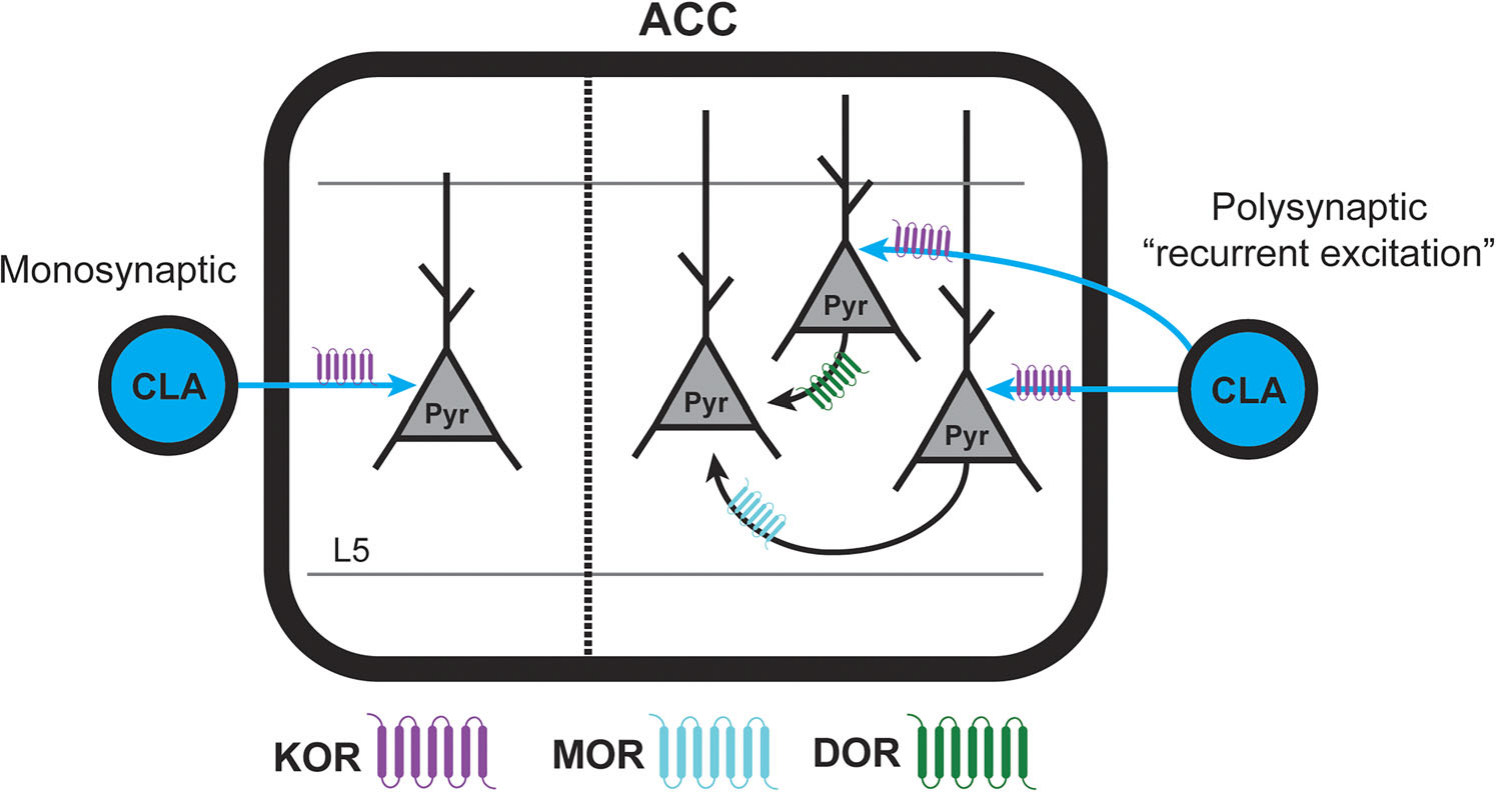
The proposed model of how excitatory synaptic transmission is modulated by opioid receptors along the CLA→ACC pathway. Left, Schematic of KOR (purple) reducing excitatory monosynaptic inputs from the CLA onto L5 ACC PYR cells. Right, Schematic of MOR (cyan) and DOR (green) reducing recurrent excitation in the ACC generated by CLA inputs.

This study characterized functional opioid receptor expression of the CLA→ACC circuit. While multiple studies have explored the relevance of this circuit in pain processing, they have not explicitly addressed the involvement of opioids and their modulation of neurotransmission. Here, we used spatial transcriptomics, brain slice electrophysiology, optogenetics, and pharmacology to determine where and how opioid receptor activation effects excitatory synaptic transmission from the CLA to L5 ACC PYR cells. We found that excitatory transmission from the CLA was reduced by KOR, MOR, and DOR agonists. Activation of KOR strongly suppressed excitatory monosynaptic inputs from the CLA, whereas MOR and DOR had very little or no effect on these monosynaptic inputs. This suggests that KOR is expressed on CLA terminals within the ACC and activation of KOR can virtually eliminate excitatory synaptic transmission from the CLA to the ACC (Fig. 7). On the contrary, MOR and DOR agonists appear to preferentially suppress polysynaptic excitation generated by the CLA onto L5 ACC PYR cells (Fig. 7); late-onset latency oEPSCs were suppressed by these agonists, while early-onset latency oEPSCs were mostly spared. Therefore, inhibition of specific aspects of the CLA→ACC circuit by all three opioid receptor types may serve to shape the temporal dynamics of network-level processing within the ACC. Our results suggest that opioid effects on circuits involved in pain and consciousness can be shaped by activation of multiple opioid receptors (Navratilova et al., 2024).

While it has been shown that KOR, MOR, and DOR transcripts are expressed along the CLA→ACC pathway, functional expression patterns and the role of opioid receptor signaling in modulating synaptic transmission within this pathway were previously unknown (Tempel and Zukin, 1987; George et al., 1994; Mansour et al., 1994; Georges et al., 1998; Wang et al., 2023). The functional neurophysiological results presented here align with previously understood expression patterns: KOR is highly expressed, and MOR is moderately expressed in the CLA, whereas DOR exhibits high expression and MOR exhibits moderate expression in the ACC. We showed multiple lines of evidence that suggest that KOR is functionally expressed on the terminals of CLA afferents in the ACC and that KOR activation dramatically reduces glutamate release from these terminals. Because we did not study KOR expression on ACC neurons explicitly and KOR activation dramatically inhibited CLA inputs to the ACC, we cannot rule out an additional role of KOR expression on local ACC neurons in further inhibiting recurrent excitation.

The mRNA transcript for MOR, *oprm1*, is highly expressed in ACC PYR cells with highest expression in L6 (James et al., 2024). Recurrent excitation driven by pyramidal–pyramidal cell interactions can be inhibited if MOR is functionally expressed on the synaptic terminals. This could explain the latency-dependent reduction of oEPSCs by MOR. Most of our evidence suggests that MOR preferentially inhibits glutamate release from recurrent excitatory circuits but not monosynaptic CLA inputs; however, Figure 5*I* showed a significant reduction in the oEPSC event slope in the presence of DAMGO which could be explained by either presynaptic or postsynaptic effects. Again, it should be noted that we observed no changes in holding current during these experiments and most potassium channels will be blocked using cesium in our internal solution, suggesting it is likely not a postsynaptic hyperpolarizing current that is mediating this effect. However, we cannot rule out a postsynaptic effect by MOR. One limitation of using the voltage-clamp technique is imperfect voltage clamp due to space clamp errors resulting in a distorted measurement of kinetics and slope conductance (Williams and Mitchell, 2008) highlighting a potential reason we observe a significant reduction in oEPSC event slope by DAMGO. More likely, there may be a low amount of MOR expression on CLA inputs resulting in an occasional small amount of presynaptic inhibition by DAMGO. Future studies identifying functional MOR expression in pyramidal cells in the ACC are needed to evaluate MOR-mediated reduction of local recurrent excitation and somatodendritic activation of G-protein–coupled inwardly rectifying potassium channels by MOR.

MERFISH data from the Allen Institute for Brain Science show robust expression of DOR in L2/3 and L6 ACC PYR cells (Yao et al., 2023). In rats, DOR activation has been shown to hyperpolarize some ACC PYR cells and inhibit EPSCs onto ACC PYR cells (Tanaka and North, 1994). If DOR is functionally expressed in ACC PYR cells, then DOR activation would reduce recurrent excitation in the ACC. This hypothesis would be supported by our data showing that DOR suppresses the late oEPSC but not the early oEPSC. Furthermore, DOR does not reduce the early oEPSC component of the compound oEPSCs suggesting that DOR suppresses later polysynaptic oEPSC events. One interesting observation is that the DOR-selective antagonist, TIPPpsi, did not reverse the oEPSC inhibition following DPDPE treatment (Fig. 2*F*). Extended Data Figure 2-2 shows that pretreatment of brain slices with TIPPpsi prevented DPDPE-mediated reduction in the CLA-evoked oEPSC onto L5 ACC PYR cells. Because DPDPE is highly selective for DOR (Goldstein, 1987; Haaseth et al., 1990), this is likely not an effect of DPDPE activating MOR or KOR receptors. This is supported by the lack of effect of DPDPE on the early oEPSC events and early-onset oEPSCs, which suggests that DPEPE effects are distinct from U69 and DAMGO (Figs. 5, 6). Furthermore, the time at which DPDPE most prominently decreases the number of oEPSC events (4–7 ms) is earlier than that of DAMGO (>7 ms; Fig. 5*B*,*C*), suggesting DPDPE and DAMGO may regulate distinct aspects of recurrent ACC circuitry. This suggests that recovery from inhibition by DOR agonists may occur more slowly than reversal of KOR- and MOR-mediated inhibition. While we do show that TIPPpsi pretreatment blocks the effect of DPDPE on oEPSC reduction, our data are consistent with DOR preferentially inhibiting recurrent excitation circuits in the ACC in a distinct and more persistent manner than KOR and MOR inhibition and is consistent with our previous observations.

In agreement with our interpretation that KOR activation reduces monosynaptic input, whereas MOR and DOR activation reduces polysynaptic input from the CLA, we observed that only U69 suppressed the oEPSC in TTX + 4-AP conditions. However, MOR activation decreased the early oEPSC event slope (Fig. 5*J*) but not the frequency of fast oEPSC events (Fig. 5*D*), suggesting MORs may affect monosynaptic CLA oEPSCs (directly or indirectly). Additionally, in the presence of TTX + 4-AP, MOR activation showed a trend toward slightly inhibiting the oEPSC; however, this did not reach statistical significance. The 4-AP (in the absence of TTX) has been shown to blunt the ability of MOR agonists to reduce GABA transmission in some contexts (Vaughan et al., 1997; Ingram et al., 1998), making a lack of reduction in the presence of 4-AP somewhat difficult to interpret. Nonetheless, even at saturating concentrations of the full agonist DAMGO, there was, at most, only modest inhibition of the early monosynaptic oEPSCs when measured using multiple approaches. This is in spite of the moderate uniform level of *oprm1* expression across all the glutamatergic CLA neurons (Fig. 1*B*,*C*). This suggests that the moderate levels of *oprm1* transcript do not result in functional MOR expression or that MOR expressed in CLA neurons is not transported to CLA terminals in the ACC in high abundance. Altogether, these data suggest that KOR may serve as a gatekeeper for information flow from the CLA into the ACC, while MOR and DOR shape the temporal dynamics of ACC processing.

Another limitation of this study is blocking NMDA receptors. NMDA receptors are known to be involved in recurrent excitation (Wang, 1999; Wang et al., 2013). MK-801, a long-lasting but not irreversible NMDA receptor antagonist, was used in our slicing and recovery aCSF solutions. In our experimental setup, it is not clear whether NMDA receptors are completely blocked during the recordings. However, our data show that even in the presence of MK-801, we were able to observe and manipulate feed-forward excitation. In the absence of MK-801, it is likely that we would see more recurrent excitation which appears to be a fundamental part of the CLA→ACC circuit (de la Torre-Martínez et al., 2025).

By activating different complements of opioid receptors, endogenous opioids have the potential to shape cortical processing. In this context, dynorphin, the KOR-preferring opioid peptide, could modulate monosynaptic transmission of CLA inputs into the ACC resulting in filtering or gating specific sensory inputs and potentially having a role in pain processing (Wang et al., 2023). Local ACC neurons, such as somatostatin inhibitory interneurons, express dynorphin (Tremblay et al., 2016) and could act on these KOR-sensitive inputs, shunting incoming information. Enkephalin, the DOR-/MOR-preferring peptide, may modulate polysynaptic transmission including slower, sustained excitatory feedback loops in the ACC, potentially modulating more cognitive or attentional aspects of sensory information. Beta-endorphin, the MOR-preferring peptide, may preferentially reduce longer-latency oEPSCs while sparing early excitatory oEPSCs. Together, dynorphins, enkephalins, and endorphins are likely to collaborate to finely tune network-level dynamics in the CLA→ACC circuit, balancing immediate sensory input processing with sustained, complex cognitive or affective processing, which could have implications for understanding pain and consciousness.

The CLA→ACC circuit has been demonstrated to play roles in pain (Xu et al., 2022; Ntamati et al., 2023; Koga et al., 2024; Zhang and Zamponi, 2024), attention (Mathur, 2014; Goll et al., 2015), and saliency (Remedios et al., 2010; Kitanishi and Matsuo, 2017; Reser et al., 2017). Therefore, some aspects of analgesic, attentive, and salient behaviors are likely to be modulated by opioids. Given the strong opioid receptor modulation within CLA→ACC circuitry, future research should seek to understand how these different receptors alter information processing in these different brain states and behavioral contexts.

## Synthesis

Reviewing Editor: Michael Michaelides, NIDA-NIH Decisions are customarily a result of the Reviewing Editor and the peer reviewers coming together and discussing their recommendations until a consensus is reached. When revisions are invited, a fact-based synthesis statement explaining their decision and outlining what is needed to prepare a revision will be listed below. The following reviewer(s) agreed to reveal their identity: Max Joffe. Note: If this manuscript was transferred from JNeurosci and a decision was made to accept the manuscript without peer review, a brief statement to this effect will instead be what is listed below.

Reviewer 1

Thank you for addressing my questions in the discussion. This is will be a very useful paper for future studies.

Reviewer 2

I am continuing a review I provided for this manuscript at the Journal of Neuroscience. In addition to the many strengths I noted during the initial review, the authors have thoughtfully addressed many of the original concerns, including SABV, expanded discussion points, clarified methods, and a concern regarding spontaneous/miniature events. Despite all of its strengths and improvements, I have a few remaining concerns that carry-over from the initial review

1) The lack of a THIPPSI block limits the interpretation of these findings. I am concerned that 1 uM DPDPE could be activating MOR and that is why the TIPPpsi block is incomplete. The affinity of DPDPE for MOR appears to be between 71 - 423 nM, with potentially lower affinity in mice than other species (see PDSP). While it is difficult to translate concentrations in brain slice studies relative to isolated cells or homogenates, it is possible that 1 uM DPDPE activates MOR in brain slices. In the prior studies (Birdsong et al. 2019), 300 nM naltrindole was used to chase effects of 1 uM DPDPE in ACC. While naltrindole is preferential for DOR over MOR, it is still fairly high affinity for MOR (0.2 - 64 nM across at least 5 studies; also see PDSP), suggesting the naltrindole concentration used previously may not have been selective and may have also blocked MOR. By contrast, TIPPSI has much lower affinity for MOR than naltirondole, raising the concern that DPDPE effects in the present studies could be related to MOR activation. I think a TIPPSI pre-treatment (or some knockout experiment) is needed to confirm that 1 uM DPDPE is selective for DOR at this synapse

2) The statistical results of main effects and interactions from 2-way ANOVAs should be provided to determine justification for performing post-tests. I only see the results of post-tests in the revised manuscript

3) (minor concern) MK801 is a long-lasting but not irreversible antagonist. Based on this, it is not clear whether NMDA receptors are blocked during the recordings. This should be plainly stated as a limitation to these studies
