# Supplementary figures and images for: Excitatory Synaptic Transmission Is Differentially Modulated by Opioid Receptors along the Claustrocingulate Pathway
Source: eNeuro. 2025 Aug 8;12(8):ENEURO.0219-25.2025. doi: 10.1523/ENEURO.0219-25.2025 (PMC12360626; doi:10.1523/ENEURO.0219-25.2025)

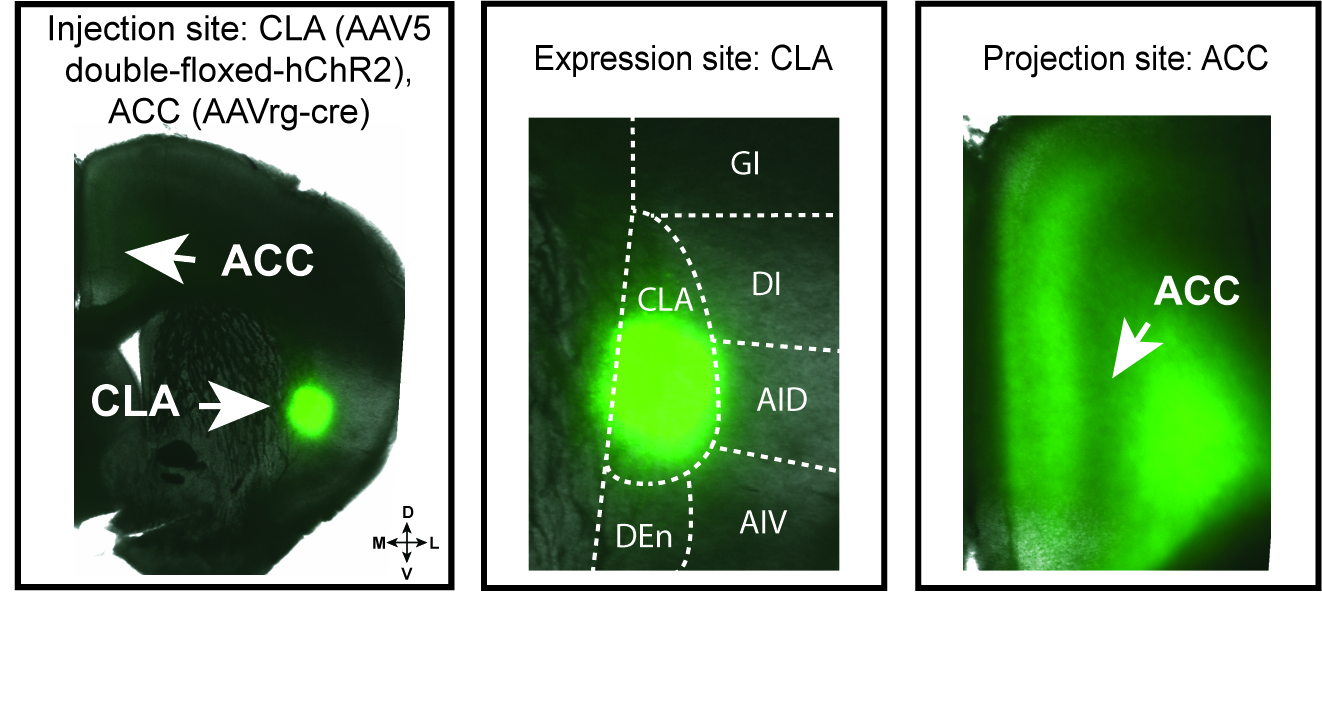

Supplement: Figure 2-1 — The ACC is innervated by the CLA and not surrounding brain regions. Cre-dependent ChR2 (AAV2-DIO-ChR2 H134R-EYFP was injected in the CLA and retrograde cre-expressing virus (AAVrg-cre) was injected in the ACC. The middle and right images are higher magnification views of the image on the left. Expression is localized to the CLA (middle). Fluorescent CLA terminals are visible in the ACC (right). Download Figure 2-1, TIF file. [file eneuro-12-ENEURO.0219-25.2025-s002.tif]

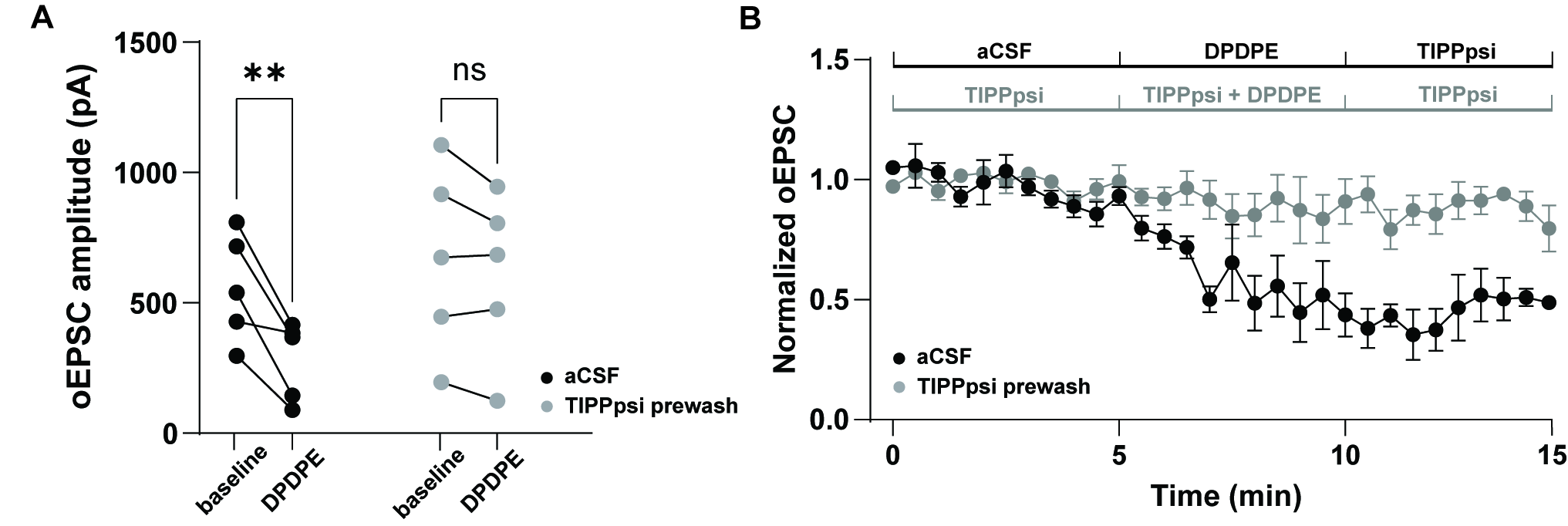

Supplement: Figure 2-2 — TIPPpsi pre-treatment blocks DPDPE reduction of CLA evoked oEPSCs onto ACC L5 PYR cells. A, Summary data showing oEPSC amplitude in baseline vs. DPDPE (1 µM) washes in aCSF (black) vs. TIPPpsi (1 µM) (gray) pretreatment conditions (oEPSC amplitude: (aCSF) baseline: 558.1 ± 93.2 pA, DPDPE: 280.3 ± 67.8 pA, N = 3, n = 5; (TIPPpsi) baseline: 667.6 ± 162.0 pA, DPDPE: 607.2 ± 143.3 pA, N = 3, n = 5; (aCSF) baseline vs. DPDPE: p = 0.0018; (TIPPpsi) baseline vs. DPDPE: p = 0.5045, two-way ANOVA with Šídák's multiple comparisons test). B, Time course of normalized oEPSC amplitude during baseline, DPDPE, and TIPPpsi perfusions in either aCSF or TIPPpsi pretreatment. A schematic is at the top of the graph illustrating the perfusions for each pretreatment condition. Download Figure 2-2, TIF file. [file eneuro-12-ENEURO.0219-25.2025-s003.tif]

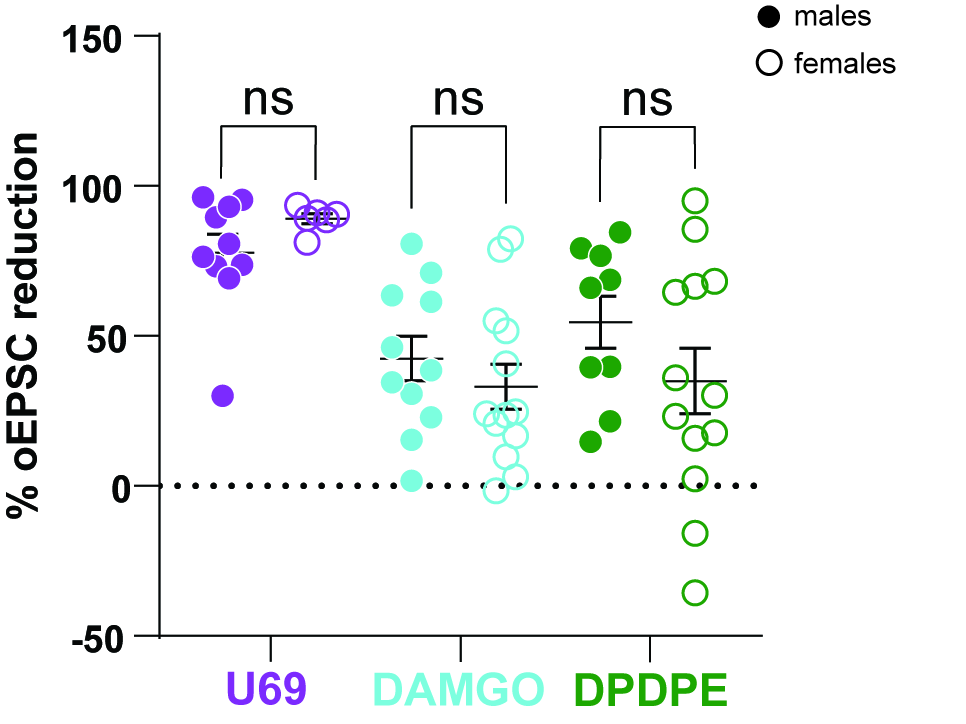

Supplement: Figure 2-3 — There are no sex differences in % oEPSC reduction across opioid receptor subtype. Summary data showing male (closed circles) vs. female (open circles) % oEPSC reduction across U69 (purple), DAMGO (cyan), and DPDPE (green) washes (% oEPSC reduction; U69 males: 77.8 ± 6.2%, U69 females: 89.1 ± 1.7%, U69 males vs. U69 females, p = 0.1882. DAMGO males: 42.5 ± 7.4%, DAMGO females: 33.1 ± 7.5%, DAMGO males vs. DAMGO females, p = 0.3888. DAMGO males: 54.6 ± 8.7%, DAMGO females: 35.0 ± 10.9%, DAMGO males vs. DAMGO females, p = 0.2071; multiple t tests analysis). Download Figure 2-3, TIF file. [file eneuro-12-ENEURO.0219-25.2025-s004.tif]
